# Experimental evidence for drought induced alternative stable states of soil moisture

**DOI:** 10.1038/srep20018

**Published:** 2016-01-25

**Authors:** David. A. Robinson, Scott B. Jones, Inma Lebron, Sabine Reinsch, María T. Domínguez, Andrew R. Smith, Davey L. Jones, Miles R. Marshall, Bridget A. Emmett

**Affiliations:** 1 NERC- Centre for Ecology and Hydrology, Deiniol Road, Bangor, UK LL57 2UW; 2 Department of Plants, Soils and Climate, Utah State University, 4820 Old Main Hill, Logan Utah, 84321-4820, USA; 3 Instituto de Recursos Naturales y Agrobiología de Sevilla (IRNAS-CSIC), Spain; 4 School of Environment, Natural Resources and Geography, Bangor University, UK

## Abstract

Ecosystems may exhibit alternative stable states (ASS) in response to environmental change. Modelling and observational data broadly support the theory of ASS, however evidence from manipulation experiments supporting this theory is limited. Here, we provide long-term manipulation and observation data supporting the existence of drought induced alternative stable soil moisture states (irreversible soil wetting) in upland Atlantic heath, dominated by *Calluna vulgaris* (L.) Hull. Manipulated repeated moderate summer drought, and intense natural summer drought both lowered resilience resulting in shifts in soil moisture dynamics. The repeated moderate summer drought decreased winter soil moisture retention by ~10%. However, intense summer drought, superimposed on the experiment, that began in 2003 and peaked in 2005 caused an unexpected erosion of resilience and a shift to an ASS; both for the experimental drought manipulation and control plots, impairing the soil from rewetting in winter. Measurements outside plots, with vegetation removal, showed no evidence of moisture shifts. Further independent evidence supports our findings from historical soil moisture monitoring at a long-term upland hydrological observatory. The results herald the need for a new paradigm regarding our understanding of soil structure, hydraulics and climate interaction.

Ecologists ~40 years ago^1^,^2^ first proposed that communities or ecosystems can be found in one of several possible alternative stable states. The theory of ASS predicts that under the same environmental conditions an ecological system can potentially exist in different, but stable states following a perturbation^3^,^4^,^5^. Mathematical models have played an important role in demonstrating the implications of the theory, which is only supported by limited empirical evidence^6^. However, much of the evidence is from analysis of historical records and rarely from manipulation experiments^7^, offering only indirect evidence which remains open to alternative explanations. Conceptually, ASS can be considered to arise from either a constant environment with shifts in variables, or alternatively from a shifting environment and change to underlying parameters. This is proposed to be the case in this work; drought eroding the resilience of the soil structure leading to a shift in soil hydraulic parameters leading to irreversible soil wetting behavior. Hence the data we present is important, offering the first experimental evidence of alternative soil moisture states induced by climate extremes, in this case drought, and changing our understanding of soil moisture dynamics.

Current predictions of climate change across Europe indicate changes to the periodicity and intensity of rainfall patterns^8^ which may increase the likelihood of extreme winter and summer weather events for Northern Europe. A consequence of this is that we are likely to see changes that increase the range of soil moisture highs and lows, with important consequences for soil mediated processes in terrestrial ecosystems such as soil respiration, net primary productivity and biogeochemical cycling. Moreover, recent research has shown that general circulation models cannot be based on atmospheric circulation alone, but must incorporate the role of land-atmosphere coupling, in particular soil moisture – temperature coupling^9^.

## Results

**Figure 1:**
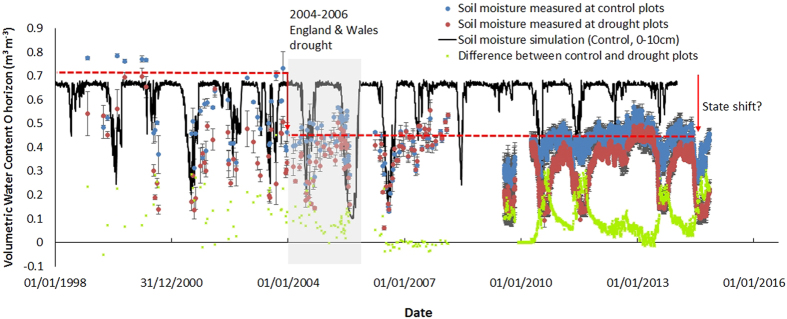
Soil moisture measurements (0–10 cm) from the climate change manipulation experiment from 1998 to date (blue dots, mean of the control sensor measurements; red dots, are the mean for drought sensor measurements, green crosses show difference in soil moisture between the control and drought treatments). The black line is a simulation of soil moisture (0–10 cm) using Hydrus 1-D^15^, based on soil hydraulic parameters determined from the control plots(Table S1), assuming a seepage face lower boundary condition (38 cm) inducing a perched water table above the weathered bedrock-soil interface (Control PW). The red dashed lines indicate a step change in winter soil moisture levels considered a consequence of the intense natural drought between 2003 and 2006.

Using a long-term drought experiment in Wales (UK)^10^ we simulated a modest, sustained reduction of summer rainfall (20–26%) over 15 years. Our site has a podzolic organo-mineral soil, with an organic horizon (~10 cm) overlying a mineral soil layer (~18 cm) over weathered fractured mudstone of the Denbigh Grits Formation (see Supplementary Fig. S1 online). Soil moisture data were collected for 15 years from the upper organic soil horizon (~10 cm). Figure 1 presents control and drought treatment averaged soil moisture data measured from within plots since 1998; the black line represents modelled soil moisture based on hydraulic properties of the control treatment. In an analysis of soil moisture data from the site early on in the experiment, 1999–2003, Sowerby *et al*.^11^ observed a divergence between the control and drought treatments. This divergence, plotted as the green markers in Fig. 1, has remained consistent with those early observations, with as much as a 0.3 m^3^ m^−3^ difference between drought and control moisture in summer, reducing to a maximum difference of 0.1 m^3^ m^−3^ in winter. However, at the beginning of 2004 a step change occurred for both the control and drought treatments, such that neither treatment rewetted in winter to the levels observed in the early years of the experiment (~0.7 m^3^ m^−3^). The most significant event at this time was the intense hydrological drought that began in England and Wales in 2003 and reached its zenith at the site in the summer of 2005^12^.

We investigated these observed changes in soil moisture retention with a combination of detailed plot measurements and modelling to understand how these changes in soil moisture occurred, with an apparent transition to new stable states for both control and drought treatments in spring 2004. The experiment tested the hypothesis that repeated summer drought shifts the soil moisture retention, reducing it compared to control. The mechanism behind this hypothesis is triggered by our theory that the organic horizon is increasingly oxidized during the drought periods, resulting in enhanced organic matter mineralization, which is usually limited by water-excess, low oxygen conditions in this wet heathland. This carbon loss would result in a degradation of the organic soil structure, lowering resilience, leading to a positive feedback, decreasing soil moisture retention capacity and increasing oxygen. If this is the case the water retention in the drought treatment should diverge below that of the control. In addition, soil respiration should increase in the drought plots as carbon is lost.

**Figure 2:**
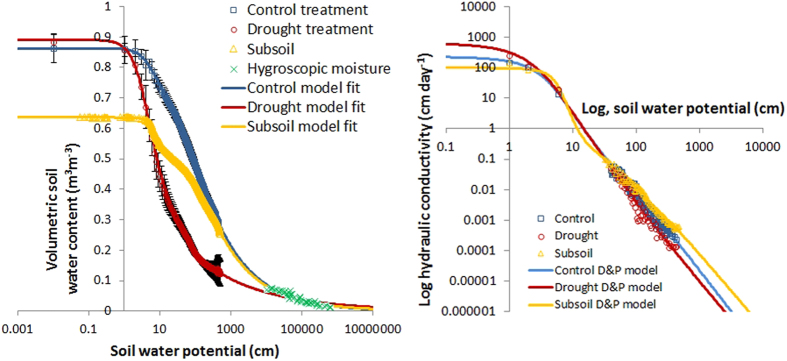
(**A**) Soil water retention curves measured in the three control and three drought plots (0–5 cm) and subsoil (10–15 cm). The data shows lower water retention in the drought treatment, considered to result from degradation of soil carbon, which substantially reduces the soils ability to retain water at a given suction compared to the control. The data are modelled using the bimodal Mualem-Durner dual porosity model^24^ and the parameters obtained were used in the Hydrus 1-D simulation model. B) The corresponding hydraulic conductivity data modelled using the Peters and Durner hydraulic conductivity model^25^.

Results in Fig. 2A confirm that the moisture retention curves between control and drought treatments have changed, resulting in a 0.29 m^3^ m^−3^ reduction in drought treatment moisture retention compared to control near saturation (−1 kPa). Recent work^13^ at the same site showed an average respiration stimulation of ~20–26% in the drought plots suggesting soil carbon degradation, supporting the hypothesis that a change in soil moisture retention capacity occurred due to a loss of soil organic matter. Further support is added from observed changes in soil bulk density which decreased significantly (t-test, p < 0.01) from 0.137 g cm^−3^ (SD = 0.069) in the control to 0.097 g cm^−3^ (SD = 0.049) in the drought plots due to carbon loss in the O-horizon (top 0–10 cm). The consequences of soil structural changes mean that after stopping the drought treatment there is unlikely to be a rapid return to initial soil moisture levels. This hysteretic response is indicative of a new stable state for soil moisture after repeated modest droughts^14^.

**Figure 3:**
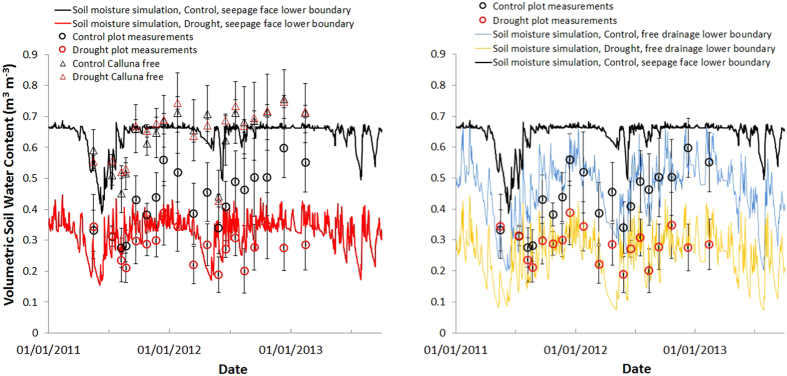
This figure indicates the existence of three stable soil moisture states possibly induced by rainfall-vegetation interaction. Independent measurements of soil moisture (0–10 cm) with fifteen measurements per treatment averaged. (**A**) Comparison of the measurements for both *Calluna* free area and plots with the Hydrus 1-D soil hydraulic model simulation, assuming a seepage face lower boundary. (**B**) The same data for the plots only but with the simulation bottom boundary condition changed to free drainage (black line of the control with seepage face shown for comparison).

The change in soil water retention capacity accounts for the differences observed between treatments, but it doesn’t explain why the control plots stopped rewetting in winter after the 2003–2006 drought period. To fully understand the impact of both experimental and natural drought on soil moisture dynamics further hydraulic measurements were made. In addition to the measurement of the soil water retention curves, hydraulic conductivity measurements and additional soil moisture measurements were conducted within the plots from 2011–2013 to provide more data to better parameterize a 1-D hydrological model^15^. Saturated hydraulic conductivity was determined based on laboratory and field measurements (K_sat_ control 238 cm day^−1^; drought 670 cm day^−1^; mineral subsoil 106 cm day^−1^) (Fig. 2B shows the unsaturated hydraulic conductivity); a list of measurements used to parameterize the model are presented in Supplementary Table S1 (online). An independent soil moisture sensor was used at the field site to measure moisture content (0–10 cm) in all the plots, and in a 0.5 m^2^ *Calluna* free area outside each plot where vegetation had been removed at the beginning of the experiment (1999), effectively allowing a third treatment to test the model against. Observation of soil close to saturation in winter in these *Calluna* free areas, but unsaturated subsoil/weathered rock below the mineral layer, led to initial model parameterization using a seepage face lower boundary condition (Figs 1 and 3A); restricting water flow and resulting in perched water in the organic horizon. The simulation worked well for both control and drought prior to the onset of the natural drought from late 2003–2006 (see Supplementary Fig S2 online), and for the *Calluna* free areas (Fig. 3A). However, when the same boundary condition was applied to the 2011–2013 control and drought plot data there was poor correspondence (Fig. 3A). The plots showed no sign of saturated conditions and so the simulation lower boundary condition was altered to free drainage, removing the perched water and improving the performance of the model to match measured plot data (Fig. 3B). Fig 3B and the *Calluna* free area in Fig 3A essentially illustrate the coexistence of three alternative stable states where the parameters are altered due to drought and presence or absence of vegetation cover.

## Discussion

These results indicate two distinct processes operating as a consequence of drought, firstly a gradual erosion of organic matter due to the sustained moderate drought, restructuring the organic layer and reducing the moisture retention characteristics in the drought treatment compared to control; this accounts for soil moisture differences observed in Sowerby *et al*.^11^. Secondly, a step change in soil moisture retention affecting both control and drought treatments following the intense natural drought period (2003–06), after which soil moisture levels never reached saturation again. The combination of measurements and modelling indicate that the bottom boundary behavior was modified during this period, changing from seepage face behavior before 2004, to free drainage after. The soil under the *Calluna* free areas did not shift, and still retained water, indicating that the presence of *Calluna* may be key to the change in boundary condition behavior. Data on root distribution supports a migration of roots down into the soil profile (see Supplementary Fig. S3 online) but not deep into the mineral layer. Though desiccation of the mineral soil by water uptake suggested by the simulation could have caused cracking. Similar structural modification of soil by *C. vulgaris* leading to soil pipe development has been observed in peat, resulting in increased drainage^16^.

Whilst alternative stable soil moisture states have been proposed to exist in savannahs^17^, this is the first manipulation experiment evidence for soils in any system. No major shift in the above ground vegetation has occurred, but the *C. vulgaris* in the drought treatment has transitioned to a degraded state with the canopy structure opening up and a decrease in the amount of C-fixing biomass observed during the experiment. Though no above ground change in vegetation species richness or abundance has occurred, the change in soil water release characteristic below ground shows that the soil has been degraded lowering the soil moisture storage capacity.

Trawling through historic data from similar sites enabled us to find comparable response patterns following drought on similar soils^18^ (see Supplementary Fig. S4 online). The data indicate that organic peat has a greater ability to buffer drought, presumably because of its greater moisture storage capacity than thinner, more vulnerable, organo-mineral soils. Though our evidence is restricted to organo-mineral soils, it has called us to question if the hydraulic modification of a lower impeding soil horizon is a more widespread phenomenon and an integral part of soil formation in young soils? Part of soil formation is often the development of a B-horizon where finer mineral materials accumulate reducing hydraulic conductivity. Could drought therefore cause an oscillation in soil moisture states as drought fractures a B-horizon over a more conductive C-horizon creating a hydraulic breach which may reseal between droughts? In time, one would assume that the thickness of the B-horizon would become such that it would overcome any vulnerability, no longer being susceptible to breach due to desiccation and so any oscillation would cease.

Wet organo-mineral soils contain considerable organic carbon stocks and have a particular vulnerability to the predicted changes of temperature and rainfall^9^. The increased oxygenation in these soils due to changing rainfall patterns could lead to the release of substantial amounts of soil organic carbon as soil respiration is enhanced under drier soil conditions, this response having potential broad implications for a positive feedback between terrestrial ecosystems and climate change^17^. Our results clearly show that both repeated modest, and intense drought eroded the resilience of these organo-mineral soils through changes in key hydraulic parameters, resulting in new stable states of soil moisture that persist after drought cessation. Failure to predict the existence of these ASS might lead to inaccuracies in the projections of carbon emissions from terrestrial ecosystems to different climate change scenarios. These projections need to be able to simulate changes in the range of soil moisture due to increasingly frequent drought events, considering the resilience of soil physical parameters as a dynamic property of the ecosystem.

## Methods

### Field site

The study was conducted at the Clocaenog climate change field site in NE Wales, UK, which has been described elsewhere^11^,^13^. The ecosystem is an upland Atlantic heathland dominated by *Calluna vulgaris* (L.) Hull. Whole-ecosystem simulation of drought and control was started in 1999 and has been running to date. Drought and control blocks were established at the site, each one with three experimental plots (4 × 5 m) randomly allocated. Fully automated retractable curtains were placed over the plots to exclude the rain from the plots from June to September annually. These produced on average a 20–26% reduction in rainfall input during the time the roofs were active over the summer. The site has podzolic organo-mineral soils, these soils have an organic rich horizon (organic matter content 89%) ~10 cm deep overlying a mineral soil layer (organic matter content of 37%) ~28 cm thick. They are classified as Ferric stagnopodzols in the Hafren Series in the Soil Survey of England and Wales^19^, and as Endoskeletic Histic Stagnic Albic Podzols in the IUSS Working Group WRB^20^ classification. The field site has intensive monitoring equipment including a meteorological station, the data from which were used as drivers in the modelling. Data for the Climoor experiment is available through the EIDC (http://eidc.ceh.ac.uk/).

### Soil moisture

This was measured at the site between 1998 and 2008 using a handheld soil moisture sensor (ThetaProbe ML2, Delta-T Devices Ltd, Cambridge), and in later years, 2008 to present, using embedded moisture sensors (TDR, Campbell Scientific Ltd, Shepshed). Additional independent measurements made between 2011 and 2013 were collected using a handheld dielectric sensor^21^ (TDR, Soil Moisture Equipment Corps, Santa Barbara, CA). Calibration of sensors was checked by comparing sensor measurements with volumetric soil moisture measurements using field soil, dried in the oven at 105 °C.

### Hydraulic measurements

Soil water release curves were measured on 250 cm^3^ soil cores, 0–5 cm deep, extracted from each plot under heather. Water release curves typically took one month to determine using the laboratory evaporation method using a hyprop^22^ (UMS, Munchen, Germany). The very dry end of the water retention curve (Fig. 2) was measured on samples using a WP4 (Decagon devices, Pullman, Washington, USA)^23^. Hydraulic conductivity was determined at low suctions (1–6 cm) with the hyprop and at high suctions (30–800 cm) in the field using a mini-disk infiltrometer (Decagon devices, Pullman, Washington, USA). Moisture release curve data was modelled using HYPRO-FIT software (UMS, Munchen, Germany) to determine hydraulic parameters using the Mualem-Durner bimodal soil water retention curve^24^ and the Peters and Durner hydraulic conductivity model^25^.

### Soil moisture model

A numerical model (Hydrus 1-D)^15^ was used to simulate soil moisture for the entire soil, from which moisture values presented for the 0–10 cm layer were extracted. The hydraulic, soil and plant parameters were measured from the field site and the driving meteorological data was measured at the field site, and where required infilled with data from a nearby weather station at Alwen^26^. The simulation was run from 01/01/1998 with initial conditions set to field capacity. The potential ET was determined from available daily precipitation, and max/min temperature data using the Hargreaves formula^27^ and the upper boundary condition was set to atmospheric with surface runoff.

## Additional Information

**How to cite this article**: Robinson, D. A. *et al*. Experimental evidence for drought induced alternative stable states of soil moisture. *Sci. Rep.* **6**, 20018; doi: 10.1038/srep20018 (2016).

## Supplementary Material

Supplementary Information
