# Supplementary material for: Experimental evidence for drought induced alternative stable states of soil moisture
Source: Sci Rep. 2016 Jan 25;6:20018. doi: 10.1038/srep20018 (PMC4726285; doi:10.1038/srep20018)
Supplement: Supplementary Information [file srep20018-s1.pdf]

Supplementary information

Experimental evidence for drought induced alternative stable states of soil moisture

David. A. Robinson, Scott B. Jones, Inma Lebron, Sabine Reinsch, María T. Domínguez, Andrew R. Smith, Davey L. Jones, Miles R. Marshall, and Bridget A. Emmett

Table S1 Hydrus 1-D model parameters used in the soil moisture simulations.

**Dual porosity hydraulic parameters**	**Porosity**	**Residual water content**	**Alpha1 (1/cm)**	**n1**<br>**-**	**Ks (cm/day)**	**w2**<br>**-**	**Alpha2 (1/cm)**	**n2**<br>**-**
Control	0.863	0.000	0.1745	2.071	238	0.768	0.0178	1.389
Drought	0.890	0.000	0.2823	1.937	670	0.288	0.0594	1.224
Subsoil	0.636	0.002	0.136	3.904	106	0.817	0.0109	1.396
**Feddes root water uptake parameters**	**PO**<br>**(cm)**	**POpt**<br>**(cm)**	**P2H**<br>**(cm)**	**P2L**<br>**(cm)**	**P3**<br>**(cm)**	**r2H**<br>**-**	**r2L**<br>**-**	
	-10	-25	-200	-600	-15000	0.5	0.1	
**Heather parameters**	**Crop height (cm)**	**LAI/surface fraction**	**Root depth (cm)**	**Interception (mm)**				
	30	1.7	10	1.5				

Figure S1: Left, a photograph of the soil horizons from the site and to the right the schematic describing the ~10cm organic horizon over the ~18cm mineral horizon over the fractured rock. Prior to the onset of natural drought late in 2003 the soil moisture was simulated using a seepage face lower boundary (see Supplementary Fig S2). Whilst after 2006 both the control and drought had to be simulated with a free drainage lower boundary (Fig 3B main text). The area with the heather removed in 1999 was simulated using the seepage face, even after the 2003-2006 drought (Fig 3A main text).

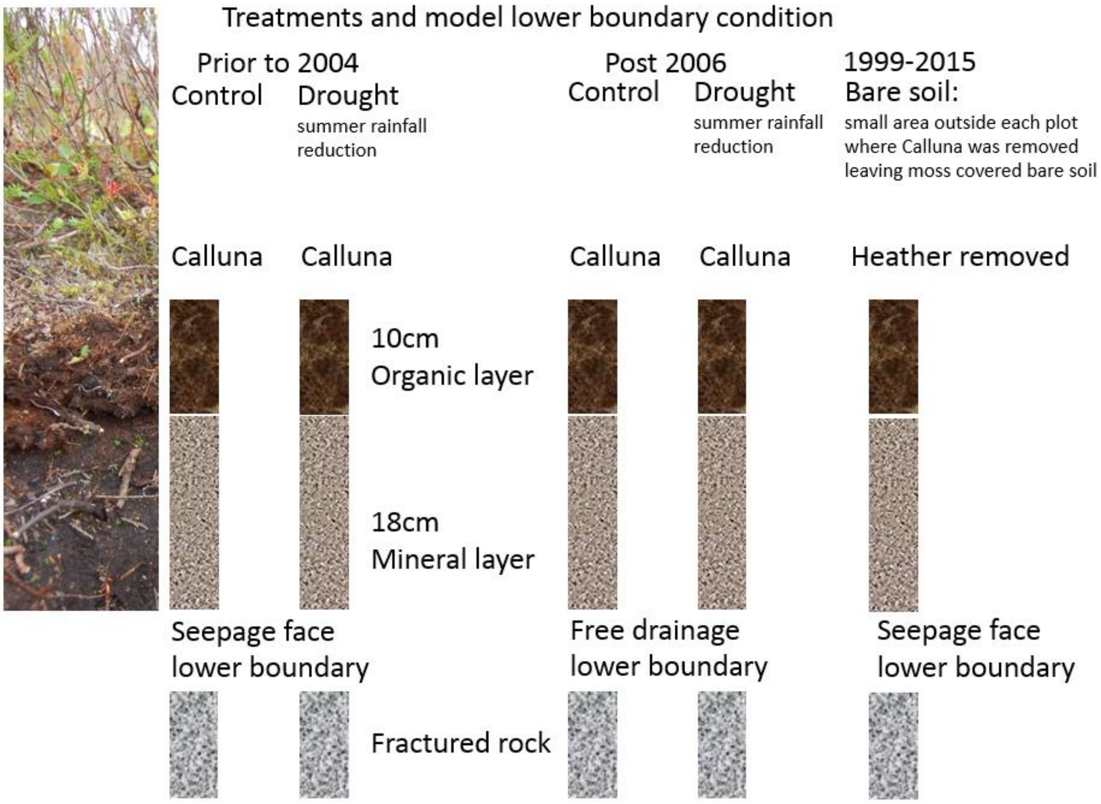

Figure S2: Soil moisture measurements and simulation for drought and control treatments prior to the natural drought from late 2003-2006. The simulation shows that the summer drought treatment of a 25% reduction in precipitation input accounts for the difference between the control and drought treatment. Both simulations assume a seepage face bottom boundary.

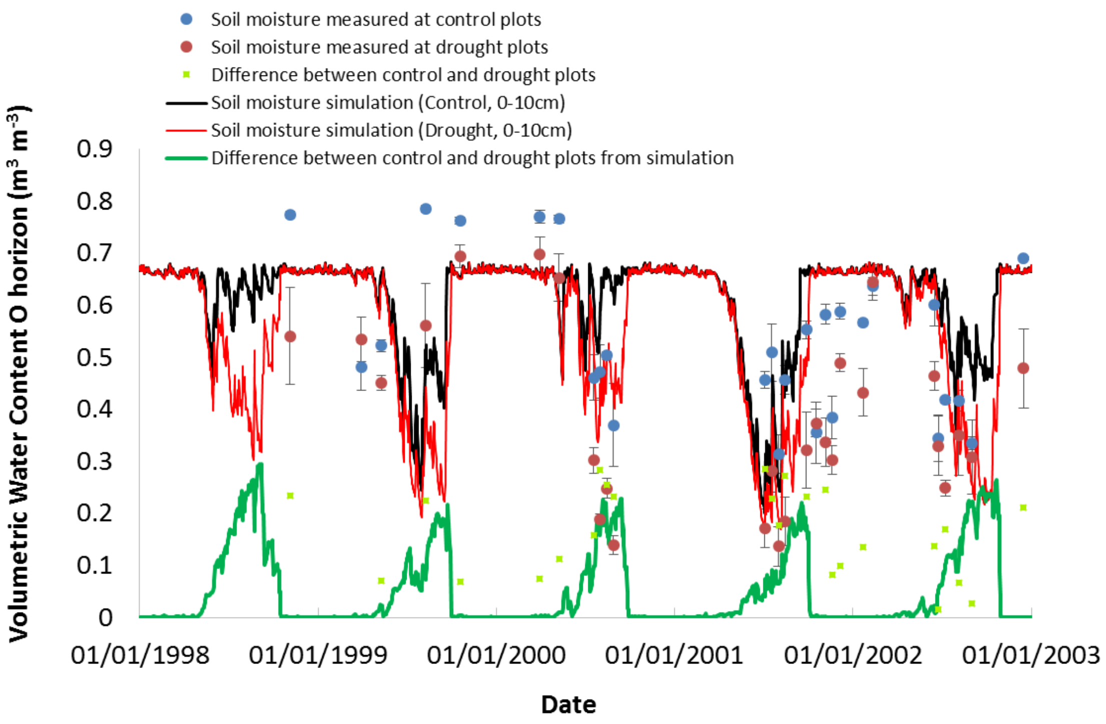

Figure S3: Fine root distribution measured in the plots in 2011; an 8 cm diameter core was taken from each plot to a depth of 10 cm. Each core was sliced into 1 cm segments, extracted roots were separated into fine (≤2 mm) and coarse (≥2 mm) size classes and washed. Root mass was determined after drying at 80 °C for 72 hours. Data presented are the percentage of total fine root biomass to a depth of 10 cm in the control (black line) and drought (red line).

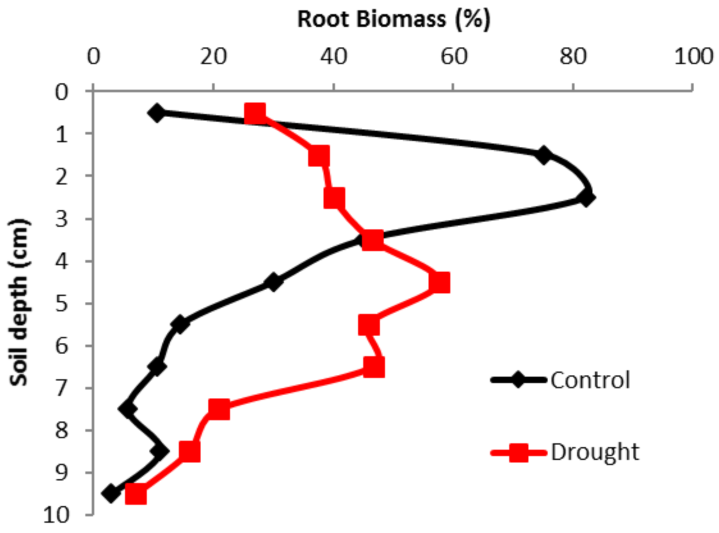

Figure S4: Soil moisture deficit for native vegetation and coniferous forest on organo-mineral podzols and peat soils; data from[1], reprinted with permission. The data are from the Plynlimon hydrological observatory about 60 miles south of the climate change field site. The podsol is the same soil series. The mire system in Fig S3C is what we expected to see with the soil drying severely in drought years (1976, 1980) and then recovering in the winter. Instead organo-mineral soils show the same response as we induced experimentally at the climate change experimental site. Drought dries the soil and the soils take time to recover, the peat soils in 3A recover quicker than the podzols in Fig 3B, with the grassland habitat in 3A recovering faster than the woodland habitat in 3A. This may indicate that peats, because they have more moisture storage are better buffered against drought, however, the peat soil in 3C with coniferous forest had not recovered by the end of monitoring in 1982 perhaps indicating a jump to a new state?

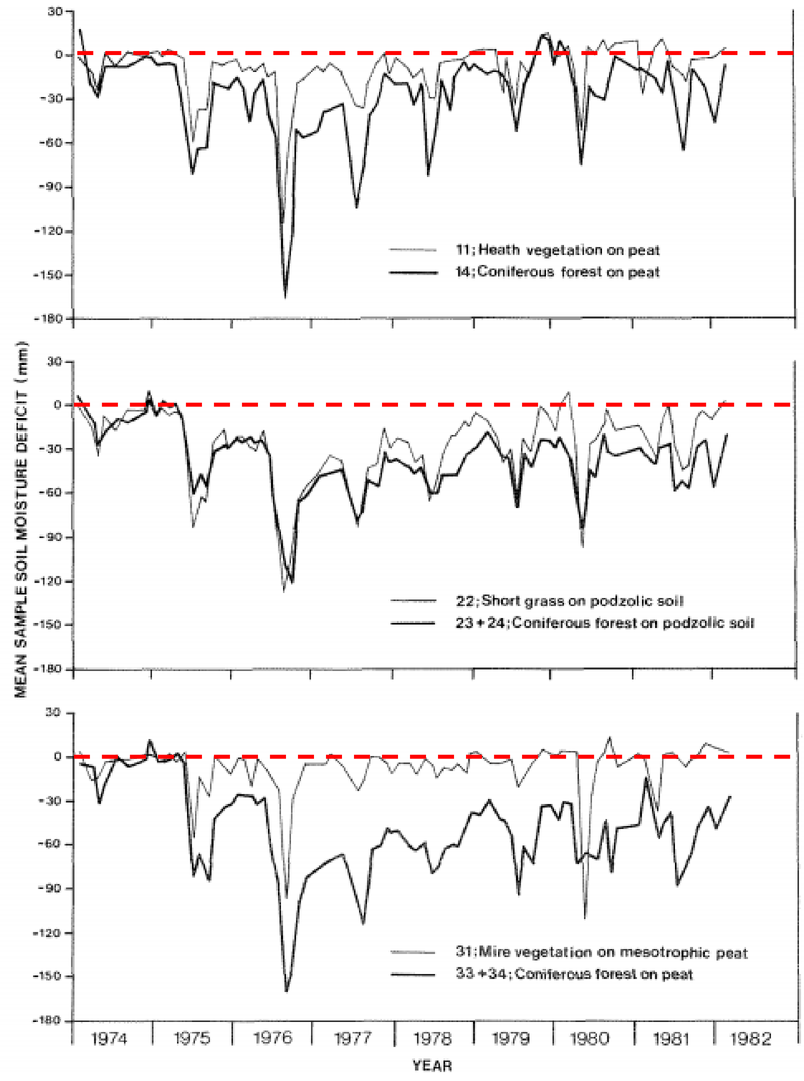

References

1 Hudson, J. The contribution of soil moisture storage to the water balances of upland forested and grassland catchments. *Hydrological sciences journal* **33**, 289-309 (1988).
